# Development and implementation of the AIDA International Registry for patients with Periodic Fever, Aphthous stomatitis, Pharyngitis, and cervical Adenitis syndrome

**DOI:** 10.3389/fped.2022.930305

**Published:** 2022-07-22

**Authors:** Francesca Della Casa, Antonio Vitale, Marco Cattalini, Francesco La Torre, Giovanna Capozio, Emanuela Del Giudice, Maria Cristina Maggio, Giovanni Conti, Maria Alessio, Benson Ogunjimi, Gaafar Ragab, Giacomo Emmi, Emma Aragona, Teresa Giani, Giuseppe Lopalco, Paola Parronchi, Farhad Shahram, Elena Verrecchia, Francesca Ricci, Fabio Cardinale, Silvia Di Noi, Rossana Nuzzolese, Riccardo Lubrano, Serena Patroniti, Roberta Naddei, Vito Sabato, Mohamed A. Hussein, Laura Dotta, Violetta Mastrorilli, Stefano Gentileschi, Abdurrahman Tufan, Valeria Caggiano, Mohamed Tharwat Hegazy, Jurgen Sota, Ibrahim A. Almaghlouth, Amr Ibrahim, Ewa Wiȩsik-Szewczyk, Burcugul Ozkiziltas, Salvatore Grosso, Micol Frassi, Maria Tarsia, Rosa Maria R. Pereira, Maged Taymour, Carla Gaggiano, Sergio Colella, Claudia Fabiani, Maria Morrone, Piero Ruscitti, Bruno Frediani, Veronica Spedicato, Henrique A. Mayrink Giardini, Alberto Balistreri, Donato Rigante, Luca Cantarini

**Affiliations:** ^1^Section of Clinical Immunology, Department of Translational Medical Sciences, University of Naples Federico II, Naples, Italy; ^2^Research Center of Systemic Autoinflammatory Diseases and Behçet's Disease Clinic, Department of Medical Sciences, Surgery and Neurosciences, University of Siena, Siena, Italy; ^3^Pediatric Clinic, University of Brescia and Spedali Civili di Brescia, Brescia, Italy; ^4^Pediatric Rheumatology Center, Department of Pediatrics, Ospedale “Giovanni XXIII”, AOU Consorziale Policlinico, Bari, Italy; ^5^Department of Life Sciences and Public Health, Fondazione Policlinico Universitario A. Gemelli IRCCS, Rome, Italy; ^6^Meyer Children's University Hospital, Florence, Italy; ^7^Department of Maternal Infantile and Urological Sciences, Sapienza University of Rome, Polo Pontino, Italy; ^8^University Department Pro.Sa.M.I. “G. D'Alessandro”, University of Palermo, Palermo, Italy; ^9^Pediatric Nephrology and Rheumatology Unit, AOU G Martino, Messina, Italy; ^10^Pediatric Rheumatology Unit, Department of Translational Medical Sciences, University of Naples Federico II, Naples, Italy; ^11^AUDACIS, Antwerp Unit for Data Analysis and Computation in Immunology and Sequencing, University of Antwerp, Antwerp, Belgium; ^12^Antwerp Center for Translational Immunology and Virology (ACTIV), Vaccine and Infectious Disease Institute, University of Antwerp, Antwerp, Belgium; ^13^Department of Paediatrics, Antwerp University Hospital, Antwerp, Belgium; ^14^Center for Health Economics Research and Modeling Infectious Diseases (CHERMID), Vaccine and Infectious Disease Institute, University of Antwerp, Antwerp, Belgium; ^15^Rheumatology and Clinical Immunology Unit, Internal Medicine Department, Faculty of Medicine, Cairo University, Giza, Egypt; ^16^Faculty of Medicine, Newgiza University (NGU), Giza, Egypt; ^17^Department of Experimental and Clinical Medicine, University of Florence, Florence, Italy; ^18^Division of Gastroenterology, Ospedali Riuniti Villa Sofia-Vincenzo Cervello, Palermo, Italy; ^19^ASST G. Pini-CTO, Department of Clinical Sciences and Community Health, Research Center for Adult and Pediatric Rheumatic Diseases, University of Milan, Milan, Italy; ^20^Rheumatology Unit, Department of Emergency and Organ Transplantation, University of Bari, Bari, Italy; ^21^Behcet's Disease Unit, Rheumatology Research Center, Shariati Hospital, Tehran University of Medical Sciences, Tehran, Iran; ^22^Department of Aging, Neurological, Orthopedic and Head and Neck Sciences, Fondazione Policlinico Universitario Agostino Gemelli IRCCS, Rome, Italy; ^23^Rare Diseases and Periodic Fevers Research Centre, Università Cattolica Sacro Cuore, Rome, Italy; ^24^Immunology Allergology Rheumatology University of Antwerp and Antwerp University Hospital, Antwerp, Belgium; ^25^Unit of Rheumatology, Azienda Ospedaliero-Universitaria Senese, Siena, Italy; ^26^Division of Rheumatology, Department of Internal Medicine, Faculty of Medicine, Gazi University, Ankara, Turkey; ^27^Rheumatology Unit, Department of Medicine, College of Medicine, King Saud University, Riyadh, Saudi Arabia; ^28^College of Medicine Research Center, College of Medicine, King Saud University, Riyadh, Saudi Arabia; ^29^International Organization for Migration, Cairo, Egypt; ^30^Department of Internal Medicine, Pulmonology, Allergy and Clinical Immunology, Central Clinical Hospital of the Ministry of National Defence, Military Institute of Medicine, Warsaw, Poland; ^31^Clinical Paediatrics, Department of Molecular Medicine and Development, University of Siena, Siena, Italy; ^32^Rheumatology and Clinical Immunology, Spedali Civili and Department of Clinical and Experimental Sciences, University of Brescia, Brescia, Italy; ^33^Rheumatology Division, Hospital das Clinicas (HCFMUSP), Faculdade de Medicina, Universidade de São Paulo, São Paulo, Brazil; ^34^Ministry of Health, Al Mounira Hospital, Cairo, Egypt; ^35^Ophthalmology Unit, Department of Medicine, Surgery and Neurosciences, University of Siena, Siena, Italy; ^36^Rheumatology Unit, Department of Biotechnological & Applied Clinical Sciences, University of L'Aquila, L'Aquila, Italy; ^37^Bioengineering and Biomedical Data Science Lab, Department of Medical Biotechnologies, University of Siena, Siena, Italy

**Keywords:** autoinflammatory diseases, international registry, personalized medicine, PFAPA syndrome, precision medicine, rare disease

## Abstract

**Objective:**

Aim of this paper is to illustrate the methodology, design, and development of the AutoInflammatory Disease Alliance (AIDA) International Registry dedicated to patients with the Periodic Fever, Aphthous stomatitis, Pharyngitis, and cervical Adenitis (PFAPA) syndrome.

**Methods:**

This is a physician-driven, non-population- and electronic-based registry proposed to gather real-world demographics, clinical, laboratory, instrumental and socioeconomic data from PFAPA patients. Data recruitment is realized through the on-line Research Electronic Data Capture (REDCap) tool. This registry is thought to collect standardized information for clinical research leading to solid real-life evidence. The international scope and the flexibility of the registry will facilitate the realization of cutting-edge study projects through the constant updating of variables and the possible merging and transfer of data between current and future PFAPA registries.

**Results:**

A total of 112 centers have already been involved from 23 countries and 4 continents starting from August 24th, 2021, to April 6th, 2022. In total 56/112 have already obtained the formal approval from their local Ethics Committees. The platform counts 321 users (113 principal investigators, 203 site investigators, two lead investigators, and three data managers). The registry collects retrospective and prospective data using 3,856 fields organized into 25 instruments, including PFAPA patient's demographics, medical histories, symptoms, triggers/risk factors, therapies, and impact on the healthcare systems.

**Conclusions:**

The development of the AIDA International Registry for PFAPA patients will enable the on-line collection of standardized data prompting real-life studies through the connection of worldwide groups of physicians and researchers. This project can be found on https://clinicaltrials.gov NCT 05200715.

## Introduction

Periodic Fever, Aphthous stomatitis, Pharyngitis, and cervical Adenitis (PFAPA) syndrome is a multifactorial autoinflammatory disorder with a still partially unknown origin (1). This is the most common periodic fever condition in children with 90% of cases occurring before the age of 5 (pediatric onset). In recent times, increasing evidence suggesting a wider expression pattern of the disease has been pointed out, with disease onset in children over 5 years (late onset) and adults more common than initially suspected (2–5).

PFAPA syndrome is characterized by high fever-episodes lasting 3–7 days recurring every 2–8 weeks; the eponymous features “aphthous stomatitis, pharyngitis, and/or cervical adenitis” are generally observed together with fever (6–14). Patients experience complete wellness between episodes, with normal growth and development when PFAPA arises during childhood. This autoinflammatory condition has a favorable long-term outcome and generally resolves as the child grows up; however, it can have a significant impact on both the child and her/his caregivers' quality of life (9, 11, 13, 15, 16).

Although, there is no certain evidence of a potential genetic basis of the disease, a significant familial clustering was shown in many studies (1). The recognition of PFAPA syndrome by clinicians is largely based on clinical features, after having excluded autoimmune, infectious, and oncologic diseases. To date, specific diagnostic criteria have been proposed by Marshall et al., later modified by Thomas et al. More recently, diagnostic criteria for adult onset PFAPA syndrome have also been developed along with Eurofever/PRINTO clinical classification criteria (7, 10, 17).

Thanks to the increasing development and use of web-related tools in medicine, patient registries have been recently adopted for research purposes in a wide range of diseases. Actually, although randomized controlled trials (RCTs) remain the gold standard for gathering evidence in clinical development, patient registries may give an answer to many kinds of questions that cannot be addressed by the traditional forms of research. This particularly true for rare diseases, for which a few numbers of patients are available for the inclusion in study protocols.

In this context, the PFAPA syndrome has been included by the AutoInflammatory Disease Alliance (AIDA) among autoinflammatory disorders to be better investigated through a dedicated International Registry. Indeed, this registry goes to add other already developed and activated registries dedicated to other autoinflammatory diseases, as non-infectious uveitis, non-infectious scleritis, Behçet's disease, monogenic autoinflammatory diseases, Still's disease, Schnitzler's syndrome, vacuoles/E1 enzyme/X-linked autoinflammatory, somatic (or VEXAS) syndrome and undifferentiated systemic autoinflammatory diseases (i.e., USAIDs) (18–20).

The focus of this paper is to highlight the development, implementation and launching of the AIDA International Registry for PFAPA syndrome, defining the methods employed, the ways in which the registry has been realized, along with the reasons, objectives, and limits associated to its deployment.

## Patients and methods

### Study design

As for other AIDA registries, this is a physician-driven, non-population- and electronic-based registry accounting for a long-term observational study. It has been conceived to gather both retrospective and prospective data from patients with PFAPA syndrome. The retrospective phase refers to real-world demographic, clinical, laboratory, instrumental and therapeutic data available at the time of the enrolment into the registry; prospective data include clinical, therapeutic, and socio-economic information accrued during the subsequent years of observation. Some variables may be referred to both retrospective and prospective phases as for cardiovascular risk, fertility, pregnancy, and post-partum period. Follow-up visits should be added at least annually and whenever treatment changes.

This registry is thought of as an observational study and data about standard routine management will be exclusively collected. Indeed, no additional laboratory or instrumental examinations are required due to the participation in the project. Similarly, none of the therapies prescribed will be determined by adherence to the study. For these reasons neither funds are provided for patient's enrolment, nor any impact on the national healthcare is determined by inclusion in the registry. As a result, the clinical management and treatments administered will be solely determined by the best standard of care and will not be affected by the AIDA project.

Any center managing patients with PFAPA syndrome could participate to the AIDA network. The only mandatory prerequisite is obtaining approval from the local Ethics Committee and appointing both a principal investigator with the charge of coordinating the study locally and at least a site investigator responsible for data collection.

Consequently, all physicians and scientists involved in the diagnostic, clinical or therapeutic management of PFAPA patients may join the AIDA Network and share information and study proposals for current and future study objectives.

### Registry objectives

The principal objective of the PFAPA registry is to collect information from a wide number of patients for research purposes. Other objectives include: I) to better define the epidemiological burden of pediatric-onset, late-onset and adult-onset PFAPA syndrome; II) to identify specific manifestations and features of late-onset and adult-onset PFAPA syndrome; III) to ascertain which is the frequency of delayed-onset PFAPA as a result of a temporally resolved early-onset PFAPA syndrome; IV) to describe any genetic abnormality in PFAPA syndrome; V) to understand the role of low-penetrance mutations in the *MEFV, TNFRSF1A, NLRP3, MVK* genes and in other autoinflammatory-associated genes in influencing PFAPA syndrome; VI) to search for specific symptoms capable of selecting subgroups of PFAPA patients; VII) to identify different triggering factors capable of inducing or influencing PFAPA attacks, including vaccinations; VIII) to detect clinical biomarkers and/or predictive factors for disease monitoring; IX) to assess the role of different therapies for PFAPA patients, including tonsillectomy, prebiotics (such as *Streptococcus salivarius* K12, *Lactobacillus plantarum* HEAL9 and *Lactobacillus paracasei* 8700:2), colchicine and other conventional disease modifying anti-rheumatic drugs (cDMARDs), small molecules and the anti-interleukin(IL)-1/anti-IL-6 biologic agents; X) to search for differences in the response to treatments between early-onset, delayed-onset and adult-onset PFAPA syndrome; XI) to identify variables capable of identifying patients more likely responsive to different therapeutic approaches; XII) to describe the socio-economic impact of the disease on patients' quality of life and national healthcare systems; XIII) to test the current clinimetric tools and assess new clinimetric instruments specific for pediatric and adult PFAPA patients; XIV) to evaluate the disease behavior during pregnancy and postpartum period; XV) to assess the long-term impact of PFAPA syndrome on the cardiovascular risk. Table 1 summarizes all these objectives for the PFAPA registry.

**Table 1:** List of objectives of the AIDA Registry dedicated to PFAPA patients.

**Main objective**	To collect information from a wide number of patients with PFAPA syndrome for research purposes
**Other objectives**	To better define the epidemiological burden of late-onset and adult-onset PFAPA syndrome
	To identify specific manifestations and features of late-onset and adult-onset PFAPA syndrome
	To ascertain which is the frequency of delayed-onset PFAPA as a result of a temporally resolved early-onset PFAPA syndrome
	To describe any genetic abnormalities occurring in PFAPA syndrome
	To understand the role of low-penetrance mutations in *MEFV, TNFRSF1A, NLRP3, MVK* and other autoinflammatory-associated genes in influencing PFAPA syndrome
	To search for specific symptoms capable of selecting subgroups of PFAPA patients
	To identify different trigger factors capable of inducing or influencing PFAPA attacks, including vaccinations
	To detect clinical biomarkers and/or predictive factors for disease monitoring
	To assess the role of different therapies for PFAPA patients, including tonsillectomy, prebiotics (such as *Streptococcus salivarius* K12, *Lactobacillus plantarum* HEAL9 and *Lactobacillus paracasei* 8700:2), colchicine and other conventional disease modifying anti-rheumatic drugs (cDMARDs), small molecules and anti-interleukin(IL)-1/anti-IL-6 biologic agents
	To search for differences in the response to treatments between early-onset, delayed-onset and adult-onset PFAPA syndrome
	To identify variables capable of identifying patients more likely responsive to the different therapeutic approaches
	To describe the socio-economic impact of the disease on patients' quality of life and national healthcare
	To test the current clinimetric tools and assess new clinimetric instruments specific for pediatric and adult PFAPA patients
	To evaluate the behavior of PFAPA syndrome during pregnancy and postpartum period
	To assess the long-term impact of PFAPA syndrome on the cardiovascular risk

*Main objective has been divided from other objectives of the Registry*.

Further objectives will be considered in relationship with future unmet needs deriving from new scientific acquisitions related to the syndrome.

### Inclusion/exclusion criteria

The primary criterion for inclusion in this registry corresponds to the fulfillment of the currently internationally accepted diagnostic and classification criteria for PFAPA syndrome. In particular, patients aged less than 5 years should fulfill the criteria proposed by Marshall et al. and revised by Thomas et al. (10); patients aged between 5 and 16 years should fulfill the Eurofever criteria for PFAPA syndrome (7); adult patients should fulfill the Eurofever criteria, or the diagnostic criteria proposed by Cantarini et al. (17). Noteworthy, this registry also includes patients with delayed-onset PFAPA syndrome after a temporally resolved early-onset one. The exclusion criteria include: the unwillingness to participate to the AIDA project; the finding of mutations on genes associated with monogenic autoinflammatory diseases, in case Eurofever criteria or diagnostic criteria are fulfilled for the corresponding monogenic disease (7, 21, 22); the identification of solid clues orienting toward the diagnosis of infectious, autoimmune diseases, malignancies, cyclic neutropenia and/or other autoinflammatory disorders.

### Online data collection

Data are collected using Research Electronic Data Capture (REDCap), an electronic data capture tool developed at the Vanderbilt University Medical Center and hosted at the Virginia Commonwealth University (Award Number UL1TR002649), which can also be used to develop patients' registries. The software is distributed at no costs and is currently used by over 5,800 Institutions in 145 Countries (23).

Investigators included into the AIDA project can log in the registry through the REDCap web-interface, insert data on the instruments of the registry and then review (and eventually complete) their own information whenever they wish. Neither principal investigators, nor site investigators are allowed to see information inserted in other centers. The electronic data entry system of the registry is in English.

While the public website of the AIDA Network (https://aidanetwork.org/en/) may be publicly accessed, the website of the registry for PFAPA syndrome (accessible at https://registries.aidanetwork.org) is hosted separately and requires personal credentials to be inserted.

Variables included in the Registry depends on the objectives of the AIDA project. In particular, the number and nature of data elements constituting the tool have been carefully selected based on the literature analysis. The time costs and the potential burden of missing data have been carefully considered before including the variables to be filled-in by the investigators. However, the widest efforts to create an all-encompassing online registry have been dedicated to this project in order to allow an evaluation of the real-world data from the many perspectives of clinical research.

In order to enhance registry feasibility and sustainability, variables included in the Registry have been distinguished into “mandatory” and “should have” information, according with the compulsory with which data have been to be collected.

### Data quality management

The maintenance of a high quality of the data entered in the Registry is essential to obtain solid results deriving from the information collected (24). As a consequence, many precautions have been adopted when creating this Registry to maximize data quality: i) quality assurance, which refers to the preliminary activities preceding data collection, to obtaining the highest quality of future data entered in the tool; ii) quality assessment, referring to periodical revisions of data included in the Registry, aimed at minimizing missing data and discrepancies; iii) quality improvement, consisting of a constant effort to keep up to date of the variables useful for future unmet needs. Of note, Site Investigators will be constantly trained to collect and enter data in the most appropriate manner.

### Ethics

The first national regulatory approval of the AIDA Project was obtained in June 2019 by the Ethics Committee of Azienda Ospedaliera Universitaria Senese, Siena, Italy (Ref. N. 14951). Later, expert centers from Europe, Middle East, North Africa, North America, and South America have approved the project and joined the AIDA Network.

This project was registered at ClinicalTrials.gov (ID: NCT05200715) and follows the principles included in the Declaration of Helsinki. In particular, patients' participation in the AIDA project is voluntary; after having received age-appropriate information sheets, the subjects enrolled (or their parents/caregivers) have to give their informed consent; minors aged ≥ 12 years have to provide their assent as a fundamental prerequisite to the enrolment. Both patients and principal investigators may withdraw their consent for the use of data for statistical analyses at all times. In case a patient withdraws the consent, no further data for that patient will be entered into the registry; moreover, if specifically requested by the patient, all previously included personal data will be completely deleted from the registry soon after the notification to the study promoter.

Patients' data are kept in accordance with the EU General Data Protection Regulations (GDPR, 2016/679/EU), or its counterparts worldwide, on the processing of personal data and protection of privacy (25).

### Statistical analysis

The statistical plan will be oriented by the specific objective of the study and by the nature of the variables analyzed from time to time. In general, the statistical plan includes general principles related to descriptive statistics and correlations, comparisons, and inferential statistics. More details will be provided as quickly as future studies will be published thanks to the data recruited in this AIDA registry.

## Results

**Figure 1:**
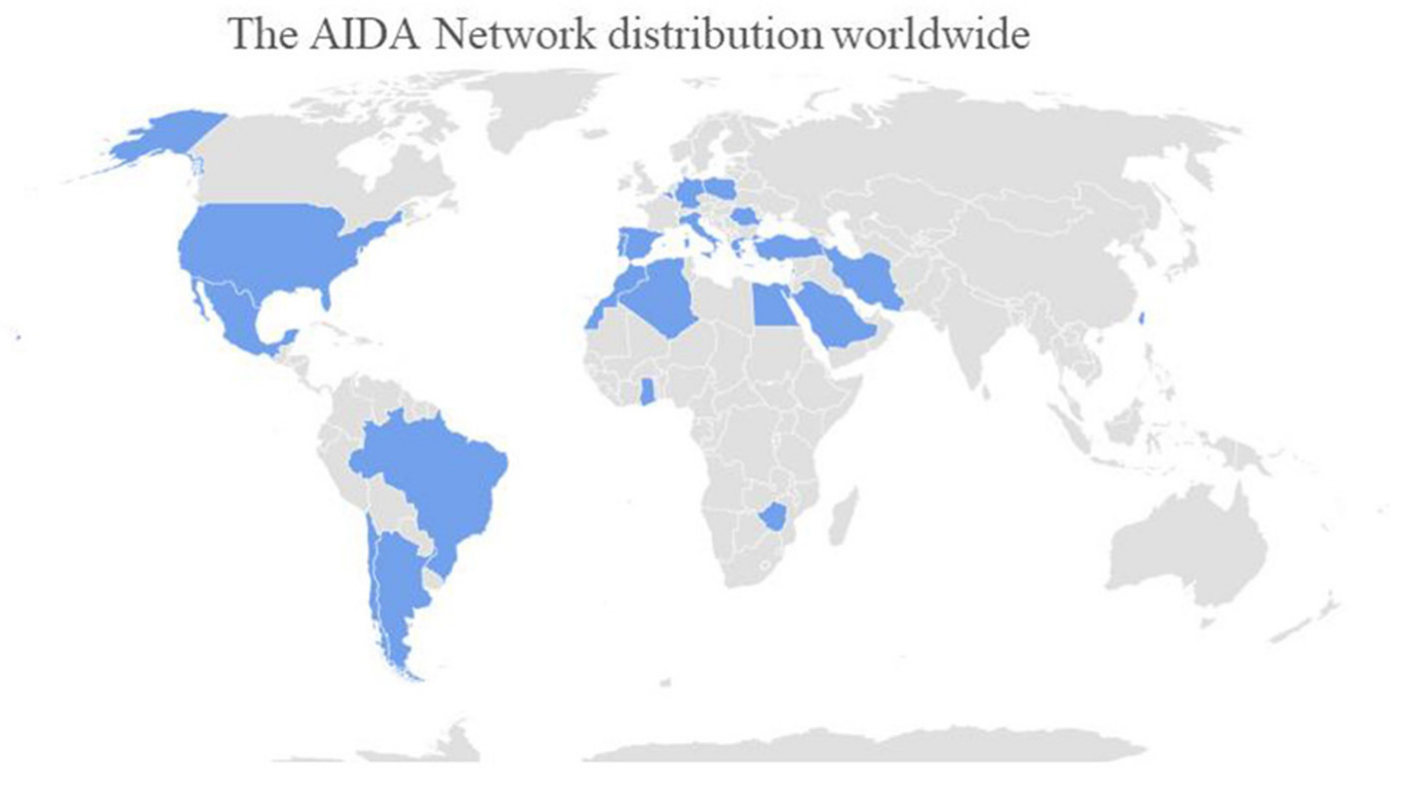
Countries involved in the AIDA Network (updated on April 6th, 2022).

The development and activation of this registry dedicated to PFAPA syndrome is *per se* the first fundamental result. Actually, this represents an essential step to obtain solid scientific information from large cohorts of patients for a very long follow-up period in different geographical contexts. Since its foundation, this network has quickly reached a wide geographic coverage. Particularly, Centers from 23 Countries (Algeria, Argentina, Belgium, Brazil, Chile, Egypt, Germany, Ghana, Greece, Iran, Italy, Lebanon, Mexico, Morocco, Poland, Portugal, Romania, Saudi Arabia, Spain, Taiwan, Turkey, United States, Zimbabwe) in 4 Continents have already joined the project sharing their knowledge and experience with autoinflammatory diseases, including PFAPA syndrome. At current, 112 centers around the world corresponding to 321 users (113 principal investigators, 203 site investigators, two lead investigators, three data managers) have taken part in the network. Figure 1 shows AIDA network distributions around the world.

The enrolment in the PFAPA syndrome registry has started on August 24th, 2020, and currently (April 6th, 2022), 149 patients (64 females/83 males/1 trans-sexual) have been enrolled. PFAPA syndrome has occurred during childhood (<16 years old) in 140 (94.6%) patients, 12 (8.6%) of which had experienced a temporary resolution during adolescence; adult-onset PFAPA syndrome was observed in 7 (4.7%) patients. In one (0.7%) patient no data were available about the age at disease onset.

### Registry development

Clinical variables constituting the registry have been chosen in order to precisely reconstruct the regular progression of a patient's clinical history in terms of diagnostic, follow-up and therapeutic course. Additional specific items have been included with the aim to face the current unmet needs. In detail, 3,856 common data elements (fields) organized into 25 instruments (forms) constitute the PFAPA registry.

Each common data element corresponds to an equal number of items that describe details about the patient's demographics, disease history, laboratory features, genetic assessment, any association with concomitant diseases, symptoms at onset, any disappearance of symptoms after tonsillectomy, any new symptom developed over time, complications including amyloidosis, cardiovascular risk, work-up exams, the impact of the disease on fertility, the disease activity during pregnancy and breastfeeding period, long-term clinical outcomes, short- and long-term response to different treatment strategies, the actual role of posology changes and drug combinations, the burden on healthcare system. The fields are organized through a branching mechanism, so that only a relatively small part of the 3,856 fields will appear during the data collection process, according to the patient's clinical history. Longitudinal data are captured through a specific follow-up instrument, which include clinical, and laboratory features as well as treatments update and socioeconomic information.

Where appropriate, general data elements are shared with other AIDA registries dedicated to different autoinflammatory diseases; other specific data elements for PFAPA syndrome have been added to describe the specific field of this disease. Both retrospective and prospective instruments include the filling-in of specific data drawn from clinical, laboratory and instrumental exams, including the observation of specific organ involvement, inflammatory indexes, and eventual development of amyloidosis. The instruments include clinical diagnostic score and criteria for PFAPA syndrome, other multifactorial autoinflammatory diseases and Eurofever scores for which the investigators must specify the eventual non-specific fulfillment (7, 10, 17, 21, 22, 26).

The instruments constituting the registry for PFAPA syndrome and the time-points at which they should refer are listed in Table 2.

**Table 2:** List of instruments (to be meant as “forms”) included in the Registry dedicated to patients with PFAPA syndrome, with the corresponding number of common data elements, time-points at which they should refer to (retrospective/prospective phase) and number of mandatory fields included.

**Instruments**	**Fields**	**Retrospective/prospective phase**	**No. of mandatory fields**
Demographics	10	Retrospective phase	4
Consents	4	Retrospective/prospective phase	2
Family history	4	Retrospective phase	0
General genetic information	5	Retrospective phase	1
Gene mutations	7	Retrospective phase	0
Diagnostic data	15	Retrospective phase	4
Features of attacks during childhood	54	Retrospective phase	3
Features of attacks at disease onset	52	Retrospective phase	3
Features of attacks up to the diagnosis	64	Retrospective phase	3
Features of attacks up from diagnosis to the time of enrolment in the AIDA Registry	64	Retrospective phase	3
Clinical diagnostic scores and criteria	13	Retrospective phase	0
Laboratory data	17	Retrospective/prospective phase	1
Cardiovascular risk	24	Retrospective/prospective phase	2
Past and current treatments	2	Retrospective phase	0
NSAIDs monotherapy - the retrospective phase	33	Retrospective phase	1
Corticosteroid monotherapy/main therapy - the retrospective phase	103	Retrospective phase	2
Colchicine treatment - the retrospective phase	77	Retrospective phase	1
Treatment with *Streptococcus salivarius* K12 - the retrospective phase	56	Retrospective phase	1
Treatment with cDMARDs (not associated to biotechnological agents) - the retrospective phase	319	Retrospective phase	6
Treatment with small molecules (not associated to biotechnological agents) - the retrospective phase	714	Retrospective phase	12
Treatment with biotechnological agents - the retrospective phase	1,146	Retrospective phase	14
Fertility and pregnancy	14	Retrospective/prospective phase	1
Disease course and treatment during pregnancies	66	Retrospective/prospective phase	1
Follow-up visits: clinical manifestations and treatment - the prospective phase	966	Prospective phase	61
Death of the patient (to open ONLY in case of patient's death)	4	Retrospective/prospective phase	0

### Patients' involvement

The role of patients has increased in the last few years, becoming progressively central in stimulating the research efforts and quality of clinical management (27). Furthermore, the role of patient advocacy organizations has fostered an important new dynamic to patient care (28). In particular, they may help in disseminating information, supporting the recruitment of patients and taking part in regulatory processes. At current, many different associations are already actively involved into the AIDA project, as the Italian Association of Periodic Fevers (A.I.F.P., Associazione Italiana Febbri Periodiche), the National Association for Rheumatologic and Rare Diseases (A.P.M.A.R.R., Associazione Nazionale Persone con Malattie Reumatologiche e Rare) and the National Rheumatic Diseases Association (A.N.M.A.R., Associazione Nazionale Malati Reumatici). The involvement of patients' associations in other countries is ongoing.

## Discussion

The AIDA registry for patients affected by PFAPA syndrome seeks to collect a large number of data from subjects experiencing early-onset disease along with the delayed-onset (for patients with a disease-onset from 5 to 16 years) and adult-onset disease. The former type of the syndrome has been more extensively studied in the past decades, thanks to the oldest description and the heavier epidemiologic burden (29, 30). Conversely, the later-onset forms are still largely unknown due to their recent identification and the rarer prevalence. In this regard, the implementation of an online network of physicians and researchers will facilitate the investigation on the lesser-known areas of PFAPA syndrome, allowing a rapid fulfillment of research goals on one hand and the achievement of robust results based on large numbers of patients on the other hand. Moreover, the prospective phase of the project will allow for a very long-term follow-up, leading to very useful findings in terms of therapeutic and clinical approach to the patient. In particular, the long-term outcomes, the rarer disease manifestations and complications, and the response to therapies in the long run will be deeply assessed. Another essential result of the registry consists in the creation of a worldwide research collaboration between different perspectives corresponding to the various specialties dealing with PFAPA syndrome, including pediatricians, rheumatologists, immunologists, gastroenterologists, dermatologists, ophthalmologists, geneticists, and internal medicine physicians. The final result of this new methodological approach to research is embodied in the spreading of knowledge and awareness of this disease with consequent implementation of diagnosis, decrease in the diagnostic delay, and hopefully optimization of clinical care of PFAPA patients.

The AIDA network is the virtual platform where the scientific community could establish the research lines on the basis of current unmet needs and the cutting-edge acquisitions in the international literature. The implementation and execution of a large international registry may overcome the limitations related to the poor availability of patients for study recruitment. Indeed, current evidence on rare diseases is almost based on small individual studies conducted by one or only a few centers (29). On the contrary, a worldwide basis of data collection will ultimately allow the resolution of old riddles about PFAPA syndrome, such as its genetic origin or influence - at least in some cases - which is currently controversial and not fully understood (1, 30, 31); the boundary line between some PFAPA subgroups and Behçet's disease, which may be very fine in some cases (16, 32); to describe uncommon symptoms and eventually individuate new subgroups of patients, who respond differently to various treatments (33); to better understand the different behaviors of the disease according to the age at disease onset. This registry may also join the research ambitions toward the achievement of a personalized medicine aimed at choosing the right treatment according to baseline features or risks to develop severe manifestations and potential long-term complications.

The registry may also provide a precious source of data to identify the impact of this disease on the cardiovascular risk and the behavior of the syndrome during pregnancy and postpartum period. Furthermore, the prospective phase of the project will provide more information about the socio-economic impact of the disease on national health systems and benefits that may result from treatments. The broad number of objectives at the root of this registry actually highlights the power of this tool in implementing the research and transforming into practical achievements the results that will derive from pooling the strengths together of worldwide reference centers.

Flexibility is one of the main features of the registry, allowing the registry to quickly improve and be implemented as the unmet needs will change and the international scientific literature will make it necessary to add new variables or remove outdated items. In addition, the flexibility combined with the ability to communicate with other current and future registries will allow the exchange and merging of information, thus further enhancing the implementation of shared research projects and the conduction of cutting-edge international studies. In this perspective, the AIDA network could represent the online platform where experts and researchers may generate ideas and initiate projects in the spirit of a full cooperation and proactive sharing of both data and results.

The AIDA registry for patients with PFAPA syndrome is burdened by the usual limitations of observational studies. Firstly, data required may be often unavailable and the frequency of missing information could be statistically relevant in some cases. This may affect some studies on one hand, but the rarity of this condition also makes any minority of data important, if the clinical question is of primary relevance. Periodic quality checks of the collected data will reduce the number of missing data, while statistical management of variables will be oriented toward optimal handling of available information. Also, entering data into the platform could take many hours, especially when the patient's medical history is particularly complex, in case of long-lasting disease, multiple treatment approaches and many posology changes. Instead, entering prospective data from follow-up visits takes 10–15 min at the most. In addition, only patients who meet the currently available diagnostic/classification criteria will be included in the registry: this will lead to the exclusion of patients with PFAPA syndrome suffering from atypical presentation. However, meeting at least one of the diagnostic/classification criteria is necessary in order to minimize the proportion of misdiagnosed patients, while avoiding over-exclusion. Patients with suspected PFAPA syndrome who do not meet the diagnostic/classificatory criteria should be included in the registry dedicated to USAIDs for future and specific investigations on atypical PFAPA syndrome.

Despite its limitations, this registry has the potential and the geographical extension to really achieve all the objectives fixed over the next years.

## Conclusion

In conclusion, an international registry dedicated to patients with PFAPA syndrome was designed and developed. It is primarily aimed at data sharing, international consultation, and dissemination of scientific knowledge related to this autoinflammatory condition. The project was thought to involve as many reference centers dedicated to autoinflammatory diseases as possible, with the opportunity to share ideas and clinical information for studies capable of providing evidence from real-world. Overcoming the scientific and clinical fragmentation about PFAPA syndrome and enhancing robust international studies represent the main reasons behind the development of this registry. The communication with other registries on PFAPA syndrome and the possibility to constantly evolve according with future acquisitions will facilitate this registry in reaching its aims.

## Ethics statement

The studies involving human participants were reviewed and approved by the Ethics Committee of Azienda Ospedaliera Universitaria Senese, Siena, Italy (Ref. No. 14951). Written informed consent to participate in this study was provided by the participants' legal guardian/next of kin.

## Author contributions

FD wrote the first draft of the manuscript. AV conceived and designed the study and revised the draft of the manuscript. DR revised the draft of the manuscript. LC conceived and designed the study and accounts for AIDA Registries Coordinator. AB is the bioengineer involved in the technical management of the platform and registries. MC, FL, GCa, ED, MMa, GCo, MA, BOg, GR, GE, EA, TG, GL, PP, FS, EV, FR, FC, SD, RNu, RL, SP, RNa, VSa, MHu, LD, VM, and SGe were involved in data recruitment in the Registry dedicated to patients with PFAPA syndrome. AT, VC, MHe, JS, IA, AI, EW-S, BOz, SGr, MF, MTar, RP, MTay, CG, SC, CF, MMo, PR, BF, VSp, and HG were included in the authorship as investigators from the top contributor centers for any of the other seven AIDA Registries. Authorship has been established based on the number of data recruited in the AIDA Registries on April 6th, 2022. All authors contributed to the article and approved the submitted version.

## Funding

Research Founding Flanders: BOg has received grant 1861219N.

## Conflict of interest

The authors declare that the research was conducted in the absence of any commercial or financial relationships that could be construed as a potential conflict of interest.

## Publisher's note

All claims expressed in this article are solely those of the authors and do not necessarily represent those of their affiliated organizations, or those of the publisher, the editors and the reviewers. Any product that may be evaluated in this article, or claim that may be made by its manufacturer, is not guaranteed or endorsed by the publisher.
